# Simulation and experiment of laser cladding layer for repairing brake disc in heavy-duty vehicles

**DOI:** 10.1098/rsos.241980

**Published:** 2025-06-04

**Authors:** Lixin Wang, Xinyu Jiang, Guang Yang, Shiyun Dong, Shixing Yan

**Affiliations:** ^1^ Hebei University of Science and Technology, Shijiazhuang, People’s Republic of China; ^2^ Academy of Armored Force Engineering, Beijing, People’s Republic of China

**Keywords:** brake discs, uneven wear, laser cladding layer, regulation mechanism, heavy-duty vehicles

## Abstract

The damage to brake discs in heavy-duty vehicles caused by uneven wear is usually repaired with milling method, which reduces their radius and accordingly shortens their service life. Laser cladding repair can maintain the radius, thereby gradually becoming a promising method for brake disc repair, but the complexity of the laser cladding process causes the cladding layer to generate defects that decrease the repair quality. Here, we studied the regulation mechanism of laser power, beam diameter and scanning speed in the cladding layer’s quality via simulation and experiment. Simulation results showed that with the increase of laser power and the decrease of beam diameter or scanning speed, the molten pool’s temperature and flow rate, and the cladding layer’s residual stress have varying degrees of increase. We then fabricated laser cladding samples (Ni60/WC on ductile iron) and acquired their micromorphology, dilution rate and microhardness, and analyzed their regulation mechanism. Experiment results exhibited that the cladding process with laser power 1400 W, beam diameter 3 mm and scanning speed 10 mm/s can make the cladding layer have a better quality. This study provides an available reference for the laser cladding repair of uneven wear in brake discs from heavy-duty vehicles.

## 1. Introduction

Brake discs in heavy-duty vehicles (armored cars, tanks) convert large amounts of kinetic energy into friction energy to decelerate or stop the vehicle’s motion [1–3], which is particularly important for their maneuverability and safety. Because of the harsh service conditions, the contact surface of brake discs frequently experiences high braking torque that produces uneven wear, which accordingly increases the risk of cracks [4–7]. For the repair of uneven wear, the commonly used method is mechanical milling, which reduces the service radius of brake discs and accordingly shortens their service life. Laser cladding technology utilizes the laser’s high-energy properties to melt the cladding powder, which fills into the uneven wear zone and then solidifies to produce a cladding layer, realizing the repair of the brake disc’s damage zone [8–11]. Therefore, this technology maintains the original radius of the brake disc, and gradually becomes a promising method for the repair of uneven wear.

For the metallic components that work in extreme conditions, the laser cladding technology has been used to repair wear damage and fatigue cracks or produce a thin coating with improved performance [12–16]. For repairing the local damage to heavy-duty train wheels, Fe/Ni/Co-based alloy powder was selected as the repair material to be melted onto damaged zones with the laser cladding method, and experiment results showed these cladding layers have significantly improved hardness and wear resistance [17]. To solve the wear damage to high tensile steel-made components, a study explored the feasibility of the repair method with the laser cladding process, and results exhibited that the AerMet-100 cladding layer has good compatibility with the substrate and significantly improved hardness [18]. Some authors have melted Ni-based alloy powder onto the brake disc’s contact surface, and the acquired cladding layer can improve its wear resistance [19]. A novel repair technique using laser cladding has been developed for repairing the fatigue cracks of ultra-high strength steel components. An experiment confirmed that the cladding process produces a high-quality metallurgical bonding layer and beneficial compressive residual stresses, accordingly improving the fatigue endurance [20]. Studies have investigated the influence of laser cladding parameters on the crack formation of the nickel alloy cladding layer, and the results showed that the parameters have a high effect on the cracking susceptibility and optimized parameters can significantly restrict the cladding layer’s crack formation [21–23]. An empirical-statistical model was adopted to predict the solidification cracking during laser cladding of Inconel 718 powder on A-286 Fe-based superalloy, and results found that the solidification cracking can be avoided by adjusting the laser cladding parameters [24]. Laser cladding technology has been utilized to apply a metal coating onto a substrate that attempts to repair the wear damage of wheel-rail components, and results showed that the cladding layer has a significantly improved tribological performance, wear resistance and cracks retardation [25]. These studies exhibit abundant achievements of laser cladding technology in resolving the wear damage and fatigue cracks of metallic parts, and produce cladding layers for improving the service performance. However, they have little relevance to repairing the uneven wear damage of brake discs in heavy-duty vehicles.

During the repair of damage to metallic parts with laser cladding technology, cladding process parameters have a significant effect on the cladding layer’s quality, and thereby many studies have focused on this topic [26,27]. The laser power significantly affects the enlargement of the heat-affected zone, and directly correlates with pitting corrosion and surface defects of the cladding layer [28]. Cladding process parameters affect the melt pool’s geometry, which accordingly influences the micromorphology and corrosion resistance of the cladding layer, and optimized parameters considerably improve the corrosion resistance [29]. A study showed that optimized laser cladding parameters make the cladding layer exhibit a high integrity and a large bonding strength, as well as producing lower friction and smoother contact surface but inferior wear resistance [30]. These studies demonstrated that the cladding layer’s micromorphology and physical properties can be improved by adjusting the cladding process parameters. Complex physical/chemical phenomena such as convective mass transfer, solidification heat transfer and stress deformation frequently occur in the laser cladding process, leading to defects including microcrack, pores, low bonding strength and high residual stress, which decreases the cladding layer quality. Therefore, to improve the repair quality of uneven wear in brake discs from heavy-duty vehicles, it is necessary to study the influence mechanism of cladding process parameters on the cladding layer’s quality.

In this study, by numerical simulation, we investigated the influence of laser cladding parameters (laser power, beam diameter and scanning speed) on the molten pool’s temperature and flow rate, and the cladding layer’s residual stress. We also investigated the effect of laser cladding parameters on the cladding layer’s micromorphology, dilution rate and microhardness. According to the acquired results, we analyzed the regulation mechanism of laser cladding parameters on cladding layer quality. This study supplies an available reference for the laser cladding repair of uneven wear in brake discs from heavy-duty vehicles.

## 2. Simulation model of laser cladding

### 2.1. Basic governing equations

Numerical simulation of the laser cladding process should follow the mass/momentum/energy conservation law, and on this basis, the theoretical equation involving thermal/stress/flow field can be deduced. The mass conversation law states that the molten pool’s mass will remain constant during the laser cladding process, and can be described as follows:


(2.1) $$\frac{\partial \rho }{\partial t}+\nabla \left(\rho \vec{v}\right)=M_{\text{s}},$$

where ρ represents the material density, $\vec{v}$ represents the velocity vector, ∇ is the divergence and $M_{\text{s}}$ is the source item. In our simulation, the pressure change of the molten pool has little impact on its fluid density, thereby the molten pool’s fluid flow can be assumed as incompressible liquid, and in this situation $\nabla (\rho \vec{v})$ = 0.

The equation of the momentum conversation law in our simulation can be expressed as follows:


(2.2) $$\rho \left(\frac{\partial \vec{v}}{\partial t}+\vec{v}\cdot \nabla \vec{v}\right)=\mu \nabla^{2}\vec{v}-\nabla p+M_{\text{s}}\cdot \vec{v}+F,$$

where ρ represents the material density, $\vec{v}$ represents the velocity vector, μ stands for the fluid viscosity and its unit is MPa⋅s, ∇ is the divergence, p is the pressure gradient force, $M_{\text{s}}$ is the source item and F represents the external force acting on the fluid.

For the energy conversation law, it can be described with the following equation:


(2.3) $$\rho \left(\frac{\partial T}{\partial t}+\vec{v}\cdot \nabla T\right)=\nabla \cdot \left(k\nabla T\right)+M_{\text{s}},$$

where ρ represents the material density, T stands for the material temperature, $\vec{v}$ represents the velocity vector, ∇ is the divergence, k stands for the thermal conductivity of material and $M_{\text{s}}$ is the source item.

### 2.2. Basic assumptions and boundary conditions

#### 2.2.1. Basic assumptions

In the laser cladding process, complex physical/chemical phenomena such as convective mass transfer, solidification heat transfer and stress deformation occur frequently, as well as the energy and matter are in a real-time change state. To simplify the simulation without decreasing accuracy, the following assumptions were proposed. (i) Fluid in molten pool is an incompressible liquid, and its flow mode is laminar flow. (ii) Materials of cladding powder and substrate are isotropic, and their thermal properties are related to the change of temperature. (iii) Volume change of substrate and cladding powder in the laser cladding process are negligible.

#### 2.2.2. Boundary conditions of thermal field

Temperature gradient change of the molten pool is an important factor in the occurrence of convective mass transfer, solidification heat transfer, stress deformation and thermal stress; accordingly, the temperature field simulation is the basis for stress/flow field simulation. Further, the temperature field generated in laser cladding is transient, and its heat transfer is achieved by thermal conduction, thermal convection and thermal radiation. Because of the high fluidity of liquid and gas, the thermal conduction is considered to occur only between solids and is calculated by the following:


(2.4) $$\phi =-\lambda A\frac{dT}{dx},$$

where ϕ stands for the heat flux, λ represents the thermal conductivity, A is the area of heat conduction, dT is the temperature difference between two points and dx is the distance between two points.

The thermal convection mainly occurs inside the fluid, and it is difficult to occur inside the solid. Therefore, the thermal convection is the most important heat transfer way inside the molten pool. Further, the ambient temperature is 22°C, and the mode of convection heat transfer is natural convection heat transfer. Under the above conditions, the equation of thermal convection can be expressed as follows:


(2.5) $$\gamma =0.023\frac{\lambda }{d}{\left(\frac{dv\rho }{\mu }\right)}^{0.8}{\left(\frac{C_{\text{p}}\mu }{\lambda }\right)}^{n},$$

where γ stands for the thermal convection coefficient, λ represents the thermal conductivity, d is the effective radius of thermal convection, v is the fluid velocity, ρ represents the material density, μ stands for the liquid viscosity, $C_{\text{p}}$ is the specific heat capacity and n=0.4/0.3 in the heating/cooling state.

For the thermal radiation, its convective heat transfer coefficient can be calculated by the following equation:


(2.6) $$Q=\varepsilon \sigma AT^{4},$$

where Q represents the radiated heat of molten pool, ε stands for the blackness coefficient of materials, σ is the Stefan-Boltzmann constant, *A* is the molten pool’s area and *T* represents the molten pool’s temperature.

#### 2.2.3. Boundary conditions of stress field

In the laser cladding process, the laser causes a significant temperature gradient difference between the substrate and the cladding powder, resulting in a significant thermal stress. There are significant differences between the substrate and the cladding powder in thermal expansion/conductivity coefficient, elastic modulus and melting point, which lead to uneven expansion and contraction. These factors result in the residual stress and morphology deformation after the solidification of the cladding layer. In the simulation of the stress field, it mainly involves the thermal stress equation, strain equation and yield equation.

According to the above analysis, the thermal stress in laser cladding process can be described with the following equation:


(2.7) $$d\sigma =d\varepsilon_{\text{o}}-d\sigma_{\text{o}}-d\varepsilon_{\text{th}},$$

where dσ represents the thermal stress, $d\varepsilon_{\text{o}}$ stands for the initial stress deformation, $d\sigma_{\text{o}}$ is the initial stress and $d\varepsilon_{\text{th}}$ is the thermal stress deformation. The strain in the laser cladding process mainly results in the thermal stress, and can be calculated by the following:


(2.8) $$d\varepsilon =d\varepsilon_{\text{c}}-d\varepsilon_{\text{p}}+d\varepsilon_{\text{th}},$$

where dε represents the strain, $d\varepsilon_{\text{c}}$ stands for the elastic stress deformation, $d\varepsilon_{\text{p}}$ is the plastic stress deformation and $d\varepsilon_{\text{th}}$ is the thermal stress deformation. The strain in the laser cladding process can be expressed as follows:


(2.9) $$\sqrt{{\left(\sigma_{\text{x}}-\sigma_{\text{y}}\right)}^{2}+{\left(\sigma_{\text{y}}-\sigma_{\text{z}}\right)}^{2}+{\left(\sigma_{\text{z}}-\sigma_{\text{x}}\right)}^{2}}\leq \sqrt{2}\sigma_{\text{s}},$$

where $\sigma_{\text{x}}$/$\sigma_{\text{y}}$/$\sigma_{\text{z}}$ represents the principal stress in the *x*/*y*/*z* axis and $\sigma_{\text{s}}$ is the yield strength of materials.

#### 2.2.4. Boundary conditions of flow field

The melting-solidification model is the basic theory of flow field simulation, and the Flow-3D software has its switch which is manipulated with the enthalpy-pore method. Further, this method treats the solid-liquid two-phase zone as a porous material and divides it into three forms. Namely, the porosity is 0 when the zone is completely solidified, the porosity is 1 when the zone is completely melted, and the porosity is 0~1 when the zone is the transition state. The equation used to describe the melting-solidification model is shown as follows:


(2.10) $$g_{1}=\left\{ \begin{array}{ll} 0 & T<T_{s}\\ \frac{T-T_{s}}{T-T_{l}} & T_{s}<T<T_{1},\\ 1 & T<T_{1} \end{array}\right.$$

where $g_{1}$ represents the liquid fraction and $T_{\text{s}}$/$T_{\text{l}}$ is the solidus/liquidus temperature.

### 2.3. Heat source model and structure model

**Figure 1.**
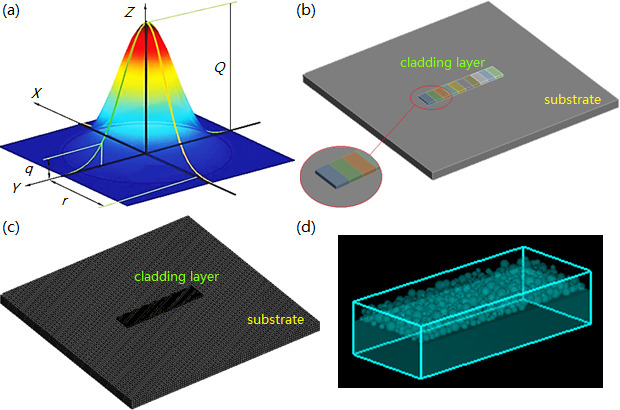
Heat source model and structure model of the simulation. (a) Gaussian heat source. (b) Structure model of thermal/stress field. (c) Mesh division of the structure model. (d) Structure model of flow field.

The Gauss heat source model was selected for the simulation of the laser cladding process. As shown in figure 1a, within a circular range, laser energy is distributed according to a Gaussian curve, with the highest energy at the central region and the lowest energy at the edge region. This characteristic is conducive to preventing excessive temperature at the overlapping zones between adjacent cladding passes, which causes the Gauss heat source model to be widely used for the simulation of the laser cladding process. The equation used to express the Gauss heat source model is shown as follows:


(2.11) $$I=\left(x,y,z\right)=\frac{2\beta P}{\pi r^{2}}exp\left[-2\frac{x^{2}+y^{2}}{r^{2}}\right],$$

where β represents the laser absorptivity, P stands for the laser power, r is the beam radius of the laser and $T_{\text{s}}$/$T_{\text{l}}$ is the solidus/liquidus temperature. *x*/*y*/*z* is the *X*/*Y*/*Z*-axis coordinates of a point. Due to the continuous movement of the laser beam along the *Y*-axis, equation (2.11) can be optimized to the following:


(2.12) $$I=\left(x,y,z,t\right)=\frac{2\beta P}{\pi r^{2}}exp\left[-2\frac{{\left(x-v\cdot t\right)}^{2}+y^{2}}{r^{2}}\right],$$

where v stands for the scanning speed and t is the scanning time.

The thermal/stress field simulation attempts to acquire the macroscopic characteristics of the melt pool, substrate and cladding layer, while the flow field simulation attempts to obtain the fluid velocity of the molten pool. Therefore, we designed the structure models of thermal/stress/flow field. For the thermal/stress field simulation, the cladding layer was set to a single layer and single pass with the structure parameters of 40 × 8 × 1 mm, as shown in figure 1b. The SOLID70 and MESH200 of ANSYS Workbench software were used to mesh the structure model. Because the temperature gradient and equivalent stress in the cladding zone are much higher than those in the substrate, the cladding zone was more finely meshed (figure 1c). For the flow field simulation, its structure model (cladding powder and substrate 100× 30 ×10 mm, figure 1d) was designed and then imported into the Flow-3D software. Because the Flow-3D software adopts the finite volume method for simulation calculation, the mesh division can be achieved by setting the grid size.

### 2.4. Material properties and laser cladding parameters

**Figure 2.**
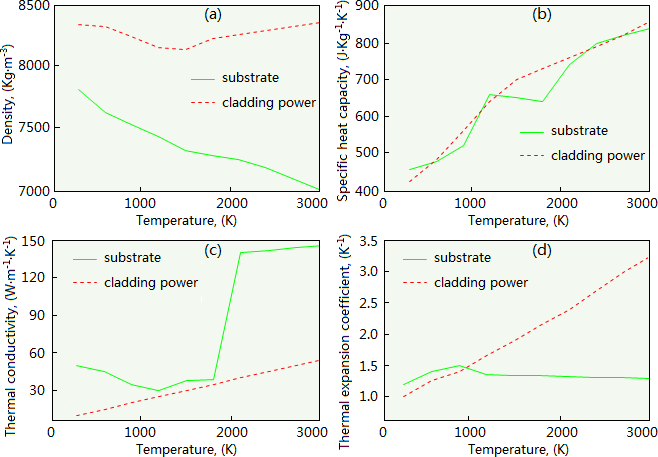
Parameters of cladding powder and substrate. (a) Density. (b) Specific heat capacity. (c) Thermal conductivity. (d) Thermal expansion coefficient.

In our simulation, the ductile iron (QT450-10) was selected as the substrate material, and the Ni60 powder was selected as the cladding material. For improving the hardness and wear resistance of the cladding layer, a WC particle with mass fraction of 10% was added to the Ni60 powder. Their main thermo-physical parameters, such as density, specific heat capacity, thermal conductivity and thermal expansion coefficient, are shown in figure 2.

Laser power (1000/1500/2000 W), beam diameter (2/3/4 mm) and scanning speed (5/10/15 mm/s) were selected as the laser cladding parameters for the simulation. The orthogonal method was adopted to combine these parameters, and accordingly, 27 parameter combinations were obtained. By the simulation, the influence of laser cladding parameters on the quality of the cladding layer was analyzed.

## 3. Simulation results and discussion

### 3.1. Influence of laser cladding parameters on thermal properties

**Figure 3.**
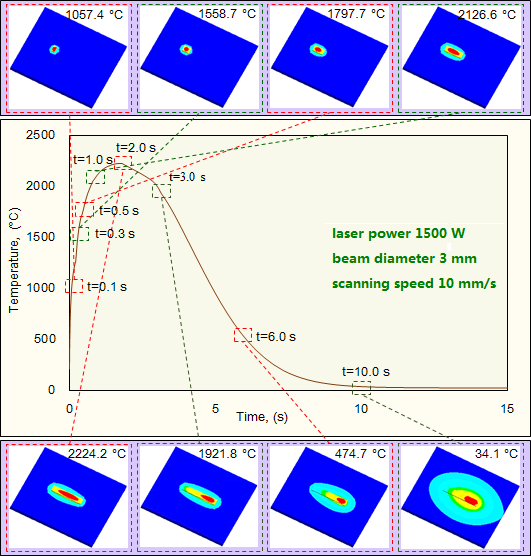
Dynamic evolution of the molten pool’s thermal properties under laser power 1500W, beam diameter 3 mm and scanning speed 10 mm/s.

After establishing the simulation model, we first conducted the thermal field simulation and measured the molten pool’s temperature to quantify the effect of laser cladding parameters on molten pool’s thermal properties. With the progress of laser cladding, the temperature of the molten pool has a significant increase. To characterize the dynamic evolution of thermal properties, take the combination of laser power 1500W, beam diameter 3 mm and scanning speed 10 mm/s as a sample. In the early stage of the laser cladding, namely the initial moment of laser irradiating on cladding powder, because of the huge temperature gradient and strong thermal convection, the temperature of the molten pool increases sharply. As shown in figure 3, the temperature reaches 1057.4°C at the moment of t = 0.1s, and achieves 1558.7°C at the moment of t = 0.3s, which exceeds the melting points of substrate (ductile iron QT450-10) and cladding powder (Ni60/WC), generating the molten pool. The temperature exhibits its maximal value 2224.2°C at the moment of t = 2.0s, and because of the gradual solidification of the molten pool, the temperature decreases sharply and shows the values of 34.1°C at the moment of t = 10.0s. The temperature exhibits the largest value in the molten pool’s central zone and dissipates to the surrounding zone, producing a high temperature gradient.

**Figure 4.**
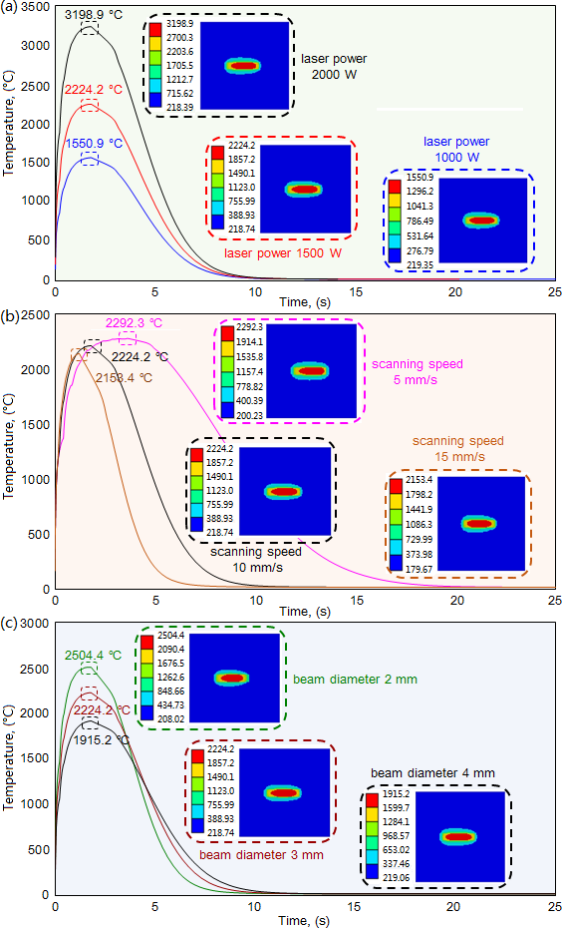
Influence of cladding parameters on molten pool’s temperature. (a) Under scanning speed 10 mm/s and beam diameter 3 mm. (b) Under laser power 1500 W and beam diameter 3 mm. (c) Under laser power 1500 W and scanning speed 10 mm/s.

To quantify the influence of laser power on the molten pool’s thermal properties, we fixed the scanning speed (10 mm/s) and the beam diameter (3 mm) and acquired the temperature corresponding to the three types of laser powers (1000/1500/2000 W). As shown in figure 4a, the molten pool’s temperature increases significantly with the increase of laser power. The temperature reaches its maximal value 1550.9°C when the laser power is 1000W, and respectively achieves its maximal values of 2224.2 and 3198.9°C when the laser power is 1500 and 2000W, exhibiting increase rates of 43.4 and 106.3%. The increase in laser power means a significant increase in laser energy density, which causes the molten pool to receive more laser energy within the same time, leading to a sharp increase in the molten pool’s temperature. Furthermore, for the three types of laser power, the time for the temperature to decrease to ambient temperature is 30.29, 25.03, and 22.09 s, respectively. That’s because the increase in laser power leads to the increase in the molten pool’s temperature, which accordingly increases the thermal conductivity of cladding material, resulting in the increase of thermal release speed in the solidification process of the cladding layer. It is noteworthy that the laser power of 1000W causes the molten pool to exhibit a small temperature, whereas the laser power of 2000W produces an excessively high temperature.

The scanning speed also generates an effect on the molten pool’s thermal properties. For analyzing this effect, we fixed the laser power (1500W) and the beam diameter (3 mm), and acquired the temperature corresponding to the three types of scanning speed (5/10/15 mm/s). As shown in figure 4b, the molten pool’s temperature decreases slightly with the increase of scanning speed. The temperature exhibits its maximal value of 2292.3°C when the scanning speed is 5 mm/s, and respectively exhibits its maximal values of 2224.2 and 2153.4°C when the scanning speed is 10 mm/s and 15 mm/s, producing the decrease rates of 2.97 and 6.06%. These slight decreases result from the increase in scanning speed, causing the decrease of laser energy received by the molten pool within the same time, which reduces the thermal conduction in adjacent zones inside the molten pool. It is noteworthy that the scanning speed can significantly affect the time when the maximal temperature appears. For the given scanning speed of 5 , 10 and 15 mm/s, the time for temperature to exhibit its maximal value is respectively 3.61, 2.00, 1.23 s. Lower scanning speed means the molten pool receives more laser energy within the same time, causing the generation of much greater temperature gradient and thermal conduction, thereby more time is required for the molten pool to reach its maximal temperature.

With the increase of beam diameter, the maximal value of the molten pool’s temperature decreases significantly. We fixed the laser power (1500W) and the scanning speed (10 mm/s), and obtained the molten pool’s temperature corresponding to the three types of beam diameter (2/3/4 mm/s). As shown in figure 4c, the temperature reaches its maximal value of 2504.4°C when the beam diameter is 2 mm, and respectively achieves its maximal values of 2224.2 and 1915.2°C when the beam diameter is 3 and 4 mm, exhibiting decrease rates of 10.47 and 23.53%. These significant decreases result from the increase of beam diameter leading to the dispersion of laser energy, which causes the decrease of the molten pool’s temperature.

### 3.2. Influence of laser cladding parameters on stress properties

**Figure 5.**
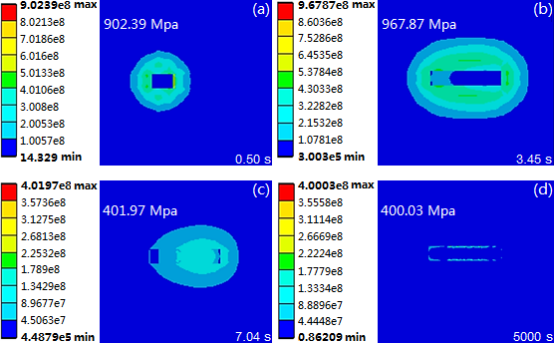
Equivalent stress nephogram of molten pool under laser power 1500W, scanning speed 10 mm/s and beam diameter 3 mm. (a) t = 0.50 s. (b) t = 3.45 s. (c) t = 0.704 s. (d) t = 5000 s.

The simulation results of the thermal field were used as thermal loads to conduct the stress field simulation, and acquired the evolution process of equivalent stress in the molten pool. This evolution process consists of without stress (initial cladding stage), sharp stress increase (cladding stage), stress reduction (solidification stage) and stress stability (complete solidification stage). Taking the molten pool produced by laser power 1500W, scanning speed 10 mm/s and beam diameter 3 mm as a sample to analyse the evolution process of equivalent stress, and its equivalent stress nephogram is shown in figure 5. In the initial cladding stage, the laser energy causes the cladding powder and the substrate to rapidly achieve their melting point and generate the molten pool. As the temperature gradient spreads in adjacent areas of molten pool, the temperature increases sharply and accordingly produces the stress with a rapid growth trend, reaching 902.39 MPa at the moment of 0.50 s (figure 5a). With the continuous input of laser energy, the molten pool’s heat-affected zone increases and accordingly the stress increases, and achieves its maximal value of 967.87 MPa at the moment of 3.45 s (figure 5b). After that, the molten pool begins to solidify which causes the decrease of stress, exhibiting a value of 401.97 MPa at a moment of 7.04 s (figure 5c). At a moment of 5000 s when the cladding layer’s temperature has decreased to ambient temperature, the stress becomes residual stress and exhibits the value of 400.03 MPa (figure 5d).

**Figure 6.**
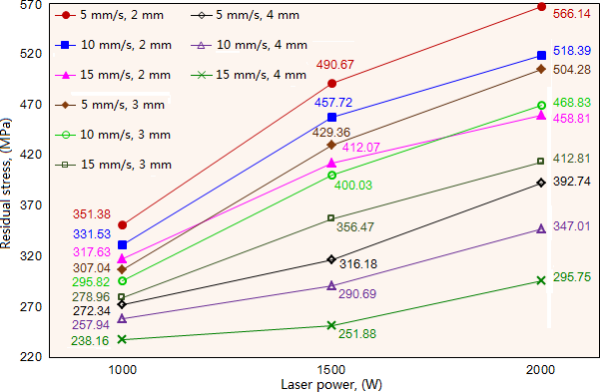
Influence of laser power on the residual stress of cladding layer under different scanning speed and beam diameter.

Laser cladding parameters have a significant effect on the residual stress of the cladding layer. Under the given scanning speeds and beam diameters, the increase of laser power causes the increase of residual stress, as shown in figure 6. That’s because the high laser power leads to the increase of the molten pool’s temperature gradient, which accordingly improves the thermal stress and enhances the distribution of residual stress in the cladding layer. When the scanning speed (10 mm/s) and beam diameter (3 mm) are fixed, the residual stress increases significantly with the increase of laser power, exhibiting the values of 295.82 , 400.03 and 468.83 MPa when the laser power is 1000, 1500 and 2000W, respectively. It is noteworthy that the increase rate of residual stress decreases gradually with the increase of laser power, exhibiting the value of 35.23% when compared with laser power 1000W, and the value of 17.20% when compared with laser power 1500W. Laser power of 2000W causes the cladding layer to exhibit excessive residual stress, especially when the beam diameter is 2 mm. This phenomenon results from the increase rate of temperature gradient reducing with the increase of laser power. When given a beam diameter of 4 mm and the three types of scanning speeds, the increase rate of residual stress increases continually with the increase of laser power. Larger beam diameter means larger heat-affected zone and under a high laser power, it is easy to generate the overburning phenomenon. In this situation, the increase of scanning speed will reduce the temperature gradient which accordingly decreases the heat transfer in different zones of molten pool, resulting in a significant enhancement of residual stress.

**Figure 7.**
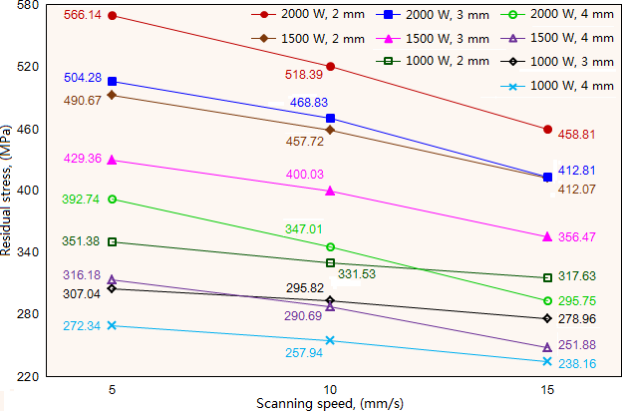
Effect of scanning speed on the residual stress of cladding layer under different laser power and beam diameter.

As shown in figure 7, under the given laser powers and beam diameter, the residual stress decreases significantly with the increase of scanning speed. When fixed laser power is 1500W and beam diameter is 3 mm, the residual stress exhibits the values of 429.18, 400.03 and 355.8 MPa when the scanning speed is 5, 10 and 15 mm/s, respectively. These results suggest that appropriate increase in the scanning speed can reduce the residual stress of the cladding layer. As the scanning speed increases, the laser energy received by the molten pool decreases, leading to the reduction in temperature gradient, which decreases the thermal stress of the molten pool and accordingly reduces the residual stress of cladding layer.

**Figure 8.**
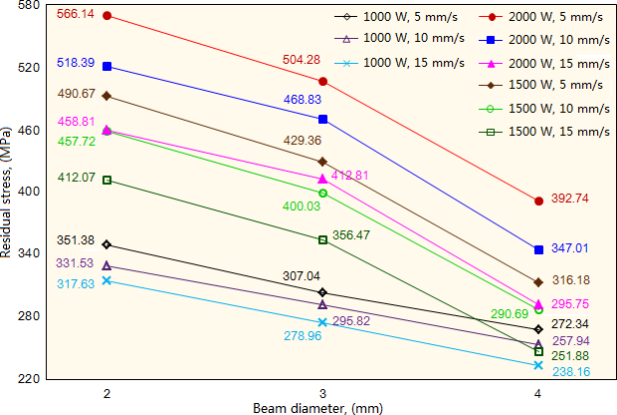
Influence of beam diameter on the residual stress of cladding layer under different laser power and scanning speed.

When given the laser power and scanning speeds, the residual stress decreases significantly with the increase of beam diameter, as shown in figure 8. Increasing the beam diameter causes the laser energy to disperse, which decreases the laser energy received by the molten pool and accordingly reduces its maximal temperature, leading to a significant decrease in residual stress. Taking the cladding layer produced by laser power 1500W and scanning speed 10 mm/s as a sample, under the three types of beam diameters (2/3/4 mm), the residual stress exhibits the values of 457.74, 400.03 and 291.15 MPa, respectively. Further, the decrease rate of residual stress has a significant increase with the increase of beam diameter, as exhibiting the value of 12.61% when compared with beam diameter 3 mm, and the value of 27.22% when compared with beam diameter 4 mm. However, under the given laser power 1000W and the three types of beam diameters, the decrease rate of residual stress has a slight variation. That’s because the low laser power causes the molten pool to produce a significant temperature gradient, which accordingly leads to the generation of powder inclusion, disrupting the variation of residual stress of the cladding layer.

### 3.3. Influence of laser cladding parameters on flow properties

During the cladding process, a moving molten pool will be generated on the substrate with the movement of the laser heat source. The flow of molten material inside the molten pool is the reason for laser energy transfer, which affects the thermal field distribution and accordingly affects the residual stress distribution. Therefore, by the flow field simulation, we analyzed the dynamic evolution of the molten pool and the influence of laser cladding parameters on the flow field’s distribution characteristics.

**Figure 9.**
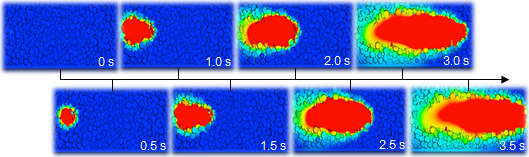
Dynamic evolution of molten pool produced by laser power 1500 W, scanning speed 10 mm/s and beam diameter 3 mm.

Take the molten pool produced by the cladding parameters of laser power 1500W, scanning speed 10 mm/s and beam diameter 3 mm as an example to analyse its dynamic evolution. As shown in figure 9, the molten pool begins to form at the moment of 0.5 s, enlarges and moves along the direction of the laser beam’s movement, and stops moving at the moment of 3.5 s. In the longitudinal direction, the molten pool’s depth decreases gradually from centre to both sides, and exhibits a tailing phenomenon.

**Figure 10.**
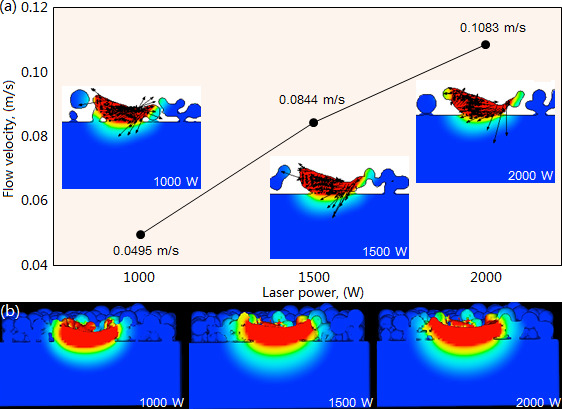
Influence of laser power on the flow velocity (a) and morphology (b) of molten pool under scanning speed 10 mm/s and beam diameter 3 mm.

The increase of laser power improves the flow velocity of the molten pool. As shown in figure 10a, under the given scanning speed 10 mm/s and beam diameter 3 mm, the flow velocity respectively exhibits its maximal values of 0.0495, 0.0844 and 0.1083 m/s when the laser power is 1000, 1500 and 2000 W. That’s because the high laser power leads to the large temperature gradient of the molten pool, which accordingly improves the flow velocity. At the moment of 1.0 s, the molten pool exhibits different morphology under the three types of laser powers (figure 10b). When the laser power is 1000W, a small amount of cladding powder fails to melt on the scanning path, because the smaller laser power produces a lower molten temperature, which leads to the incomplete melting of cladding powder. When the laser power is 1500 and 2000W, the cladding powder is fully melted, and with the increase of laser power, the molten pool’s depth/width has a significant increase.

**Figure 11.**
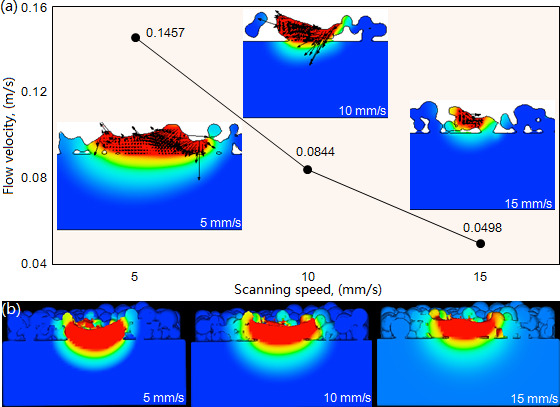
Effect of scanning speed on the flow velocity (a) and morphology (b) of the molten pool under laser power 1500W and beam diameter 3 mm.

The flow velocity of the molten pool decreases significantly with the increase of laser power. Under the laser power 1500W and beam diameter 3 mm, as shown in figure 11a, the flow velocity respectively exhibits its maximal values of 0.1457, 0.0844 and 0.0498 m/s when the scanning speeds are 5, 10 and 15 mm/s. The significant decreasing results from the increase of scanning speed reduces the laser energy received by the molten pool within an equal time, leading to the reduction in temperature gradient and accordingly decreasing the flow velocity. As shown in figure 11b, at the moment of 1.0 s, the molten pool exhibits different morphology under the three types of scanning speed. With the increase of scanning speed, the molten pool’s depth and width decrease significantly. It is noteworthy that the scanning speed of 15 mm/s causes the molten pool to produce a small dilution rate, which reduces the bonding strength between cladding layer and substrate, and also causes the cladding layer to exhibit powder inclusions.

**Figure 12.**
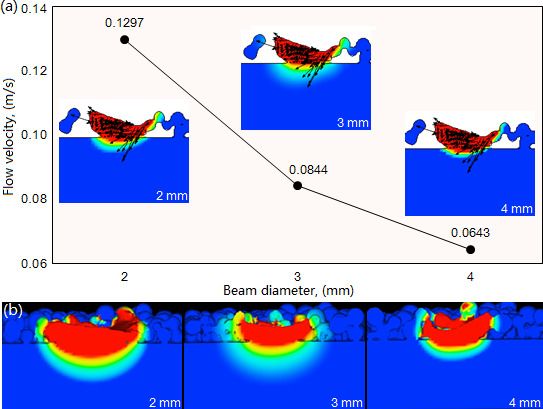
Influence of beam diameter on the flow velocity (a) and morphology () of the molten pool under laser power 1500W and scanning speed 10 mm/s.

The beam diameter has a significant effect on the flow velocity of the molten pool, as its increase causes the flow velocity to decrease significantly. Under the laser power 1500W and scanning speed 10 mm/s, as shown in figure 12a, the flow velocity respectively exhibits its maximal value of 0.1297, 0.0844 and 0.0643 m/s when the beam diameter is 2, 3 and 4 mm. The increase of the beam diameter leads to the dispersion of laser energy, which causes the decrease of the molten pool’s temperature gradient and accordingly reduces the flow velocity. As shown in figure 12b, at the moment of 1.0 s, the molten pool exhibits different morphology under the three types of beam diameter. With the increase of beam diameter, the molten pool’s depth and width have a slight decrease. It is noteworthy that when the beam diameter is 2 mm, the molten pool has an excessive depth that results in a great dilution rate. When the beam diameter is 4 mm, the molten pool has an insufficient depth which leads to a small dilution rate, reducing the bonding strength between cladding layer and substrate.

## 4. Fabrication and experiment test of cladding layer

### 4.1. Sample fabrication of cladding layer

Simulation results showed that the laser power 1000W brings out insufficient molten pool temperature and cladding layer powder inclusion, whereas the laser power 2000W produces the excessive temperature and high residual stress. The beam diameter of 2 mm causes the molten pool to generate an excessive dilution rate, whereas the beam diameter of 4 mm leads to a small dilution rate that decreases the bonding strength. The scanning speed 15 mm/s makes the molten pool generate a small dilution rate and the cladding layer exhibit powder inclusion. Therefore, we selected the scanning speed of 10 mm/s, the beam diameter of 3 mm, and the laser power of 1200/1300/1400/1500W to fabricate the cladding layer samples. For comparison, we also selected the scanning speed of 5/15 mm/s and the beam diameter of 2/4 mm.

**Figure 13.**
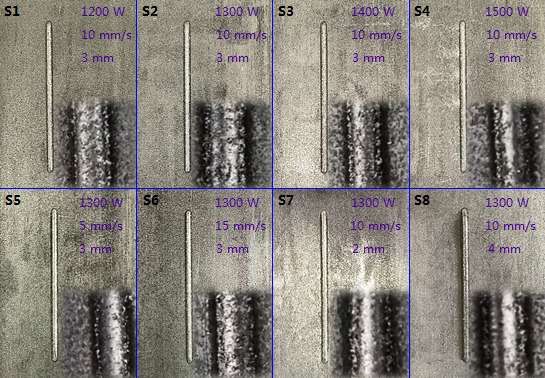
Samples of cladding layer fabricated under different cladding parameters. Inserts show the specific morphology of the cladding layer. The length of these samples is 80 mm.

After determining the cladding parameters, the laser cladding equipment HWL-R6000AW (Huawei, China) was used to fabricate the cladding layer. It has the maximal power of 6000W, adopts the coaxial powder feeding and uses the Ar as protective gas. The ductile iron (QT450-10) was used as the substrate, and Ni60 was used as the cladding powder. In addition, the WC particle (10%, mass fraction) was added to the Ni60 power for enhancing the wear resistance of the cladding layer. Before the fabrication, the cladding powder was placed on a drying oven with a temperature of 80°C for over 12 h to remove the moisture. According to the above descriptions, eight types of samples were fabricated (figure 13). For these samples, we tested their micromorphology, dilution rate and microhardness, to analyze the influence mechanism of cladding parameters on the cladding layer quality.

### 4.2. Micromorphology of cladding layers

**Figure 14.**
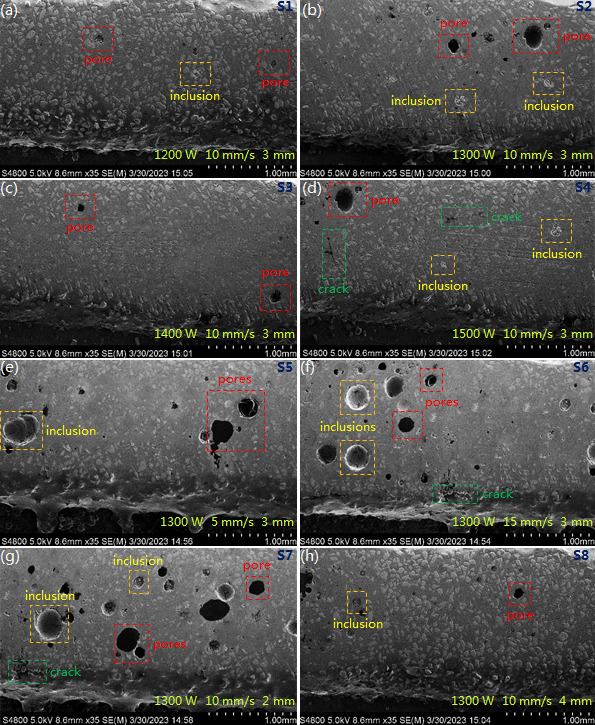
SEM images of cladding layers fabricated under different laser cladding parameters. (a/b/c/d) 1200/1300/1400/1500W, 10 mm/s and 3 mm. (e/f) 1300W, 3/15 mm/s and 3 mm. (g/h) 1300W, 10 mm/s and 2/4 mm.

The fabricated samples of cladding layer were cut to the size of 2 × 2 cm, polished with sandpapers (800#, 1500#, 2500#, 4000# and 5000#) and diamond abrasive, and rinsed with alcohol several times to remove abrasive dust. According to the similar method described in previous literature [31–33], the prepared samples were observed with a scanning electron microscope (SEM, Hitachi S-4800N, Hitachi Corp., Japan) at an accelerating voltage of 5.0 kV. By analyzing the saved SEM images of cladding layers (figure 14), defects (microcrack, pore and powder inclusion) and dilution rate can be acquired.

Viewing from the cladding layers fabricated by the scanning speed 10 mm/s, beam diameter 3 mm, and the laser power 1200W (figure 14a), 1300W (figure 14b), 1400W (figure 14c) and 1500W (figure 14d), they have relatively good morphology with very few pores, microcracks and powder inclusions. Generally, the cladding layer produced by lower laser power has more significant pores and powder inclusions. That’s because the decrease in laser power causes the reduction of temperature gradient, which slows down the escape of bubbles in the molten pool and accordingly results in more pores formed in cladding layer inclusions. In addition, the decrease in temperature gradient means more cladding powder is unable to melt completely, which leads to the powder inclusions of cladding layer. Obvious microcracks appear in the cladding layer fabricated by the laser power 1500W, scanning speed 10 mm/s and beam diameter 3 mm (figure 14d). The increase in laser power causes the increase of thermal stress, which transforms into the residual stress during the solidification process of the molten pool, resulting in the generation of microcracks.

**Figure 15.**
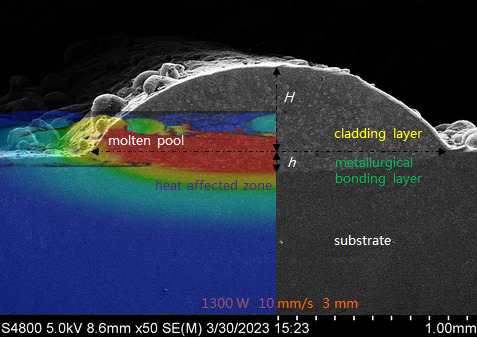
Cross-section morphology of cladding layer fabricated under laser power 1300W, scanning speed 10 mm/s and beam diameter 3 mm, and molten pool morphology acquired by simulation with the same cladding parameters.

Viewing from the cladding layers fabricated by the laser power 1300W, beam diameter 3 mm and scanning speed 10 mm/s (figure 14b), 5 mm/s (figure 14e) and 15 mm/s (figure 14f), we found that improving or decreasing the scanning speed makes the cladding layer generate more pores. Under the same laser power and beam diameter, decreasing the scanning speed causes the gasification of the molten pool that forms more bubbles, and parts of them fail to escape before the solidification of the molten pool. Increasing the scanning speed significantly reduces the duration of the molten pool, which makes it difficult for bubbles to escape before the solidification. With the increase of scanning speed, the powder inclusions of the cladding layer become more significant (figure 14f), because this increase causes the reduction in laser energy received by cladding powder, impeding the complete melting of cladding powder. The above analyses lead to a conclusion that appropriate laser cladding parameters play an important role in the cladding layer’s quality.

Viewing from the cladding layers fabricated by the laser power 1300W, scanning speed 10 mm/s, and beam diameter 3 mm (figure 14b), 2 mm (figure 14g) and 4 mm (figure 14h), we found that reducing the beam diameter causes the cladding layer to generate more significant pores and microcracks. Under the same laser power and scanning speed, decreasing the beam diameter significantly increases the laser’s energy density, which leads to an overburning phenomenon at the molten pool’s central zone due to the high temperature gradient, as well as the powder inclusions at the molten pool’s edge zone due to the insufficient melting of cladding powder. Further, with the increase of beam diameter the pores in cladding layer have a significant decrease (figure 14b,h). Accordingly, it can be concluded that appropriate laser cladding parameters can significantly decrease the defects in the cladding layer.

The laser cladding parameters also have a significant effect on dilution rate. Taking the cladding layer fabricated under laser power 1300W, scanning speed 10 mm/s and beam diameter 3 mm as an example, we compared its morphology with the molten pool morphology acquired by simulation, as shown in figure 15. High similarity in the morphology confirms the availability and accuracy of simulation. In addition, by quantitatively characterizing the height of the cladding layer and metallurgical bonding layer, the dilution rate can be calculated via the following equation:


(4.1) $$\eta =\frac{h}{h+H},$$

where η represents the dilution rate, h is the height of the metallurgical bonding layer, H is the height of the cladding layer. As shown in table 1, the height/width of the cladding layer increases with the increase of laser power, the decrease of scanning speed and beam diameter. When given the same scanning speed and beam diameter, the dilution rate increases significantly with the increase of laser power. The increase in laser power means the molten pool receives more laser energy, which means the increase in height/width of the metallurgical bonding layer is larger than that of the cladding layer, leading to the increase of dilution rate. When given the same laser power and beam diameter, the dilution rate increases with the increase of scanning speed. That’s because the increase in scanning speed reduces the laser energy received by the molten pool, leading to the height reduction of both cladding layer and metallurgical bonding layer. In addition, the cladding layer has a much greater reduction degree than the metallurgical bonding layer, causing the increase of dilution rate. When under the same laser power and scanning speed, the dilution rate reduces with the increase of beam diameter. This increase results from the small beam diameter concentrating the laser energy, which causes the cladding power to generate significant gasification to increase the height of the metallurgical bonding layer.

**Table 1.** Parameters of cladding layer and metallurgical bonding layer, and dilution rate.

sample	laser power (W)	scanning speed (mm/s)	beam diameter (mm)	height of cladding layer (mm)	width of cladding layer (mm)	height of metallurgical bonding layer (mm)	dilution rate (%)
S1	1200	10	3	0.55	2.01	0.07	11.29
S2	1300	10	3	0.57	2.08	0.08	12.31
S3	1400	10	3	0.62	2.24	0.09	12.68
S4	1500	10	3	0.71	2.52	0.11	13.41
S5	1300	5	3	0.84	2.40	0.08	8.70
S6	1300	15	3	0.54	1.97	0.09	14.29
S7	1300	10	2	0.86	2.63	0.18	17.30
S8	1300	10	4	0.52	1.82	0.04	7.14

### 4.3. Microhardness of cladding layers

**Figure 16.**
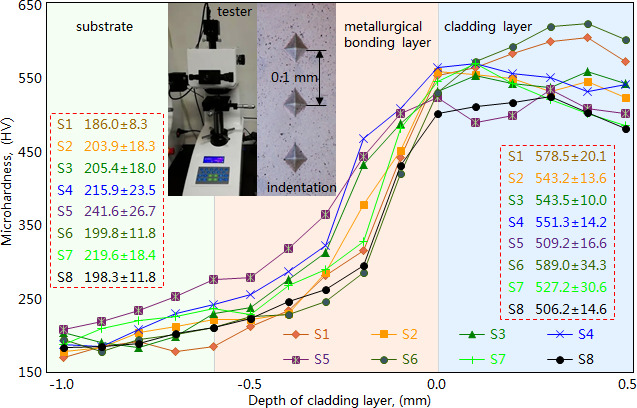
Microhardness of cladding layers at different depths. S1/S2/S3/S4, under 1200/1300/1400/1500W, 10 mm/s and 3 mm; S5/S6, under 1300W, 3/15 mm/s and 3 mm; S7/S8, under 1300W, 10 mm/s and 2/4 mm. Number of measurement is 8.

Microhardness of the fabricated cladding layers was measured with a microhardness tester (HV-100, Mitutoyo Co., Japan) under the load 200 g and holding time 15 s. The microhardness was measured three times at intervals of 0.1 mm along the direction of the cladding layer’s depth, and their average value was adopted as the microhardness. After the measurement, the microhardness of cladding layers at different depths can be acquired, as shown in figure 16. The microhardness of the cladding layer and substrate exhibits slow changing values, whereas the metallurgical bonding layer has significant increasing values. Affected by the received laser energy, the metallographic structure of these metallurgical bonding layers has significant variations, leading to the considerable increase in microhardness. For each metallurgical bonding layer, the laser energy received at different depths shows a significant difference, which results in the different value of microhardness.

In addition, we obtained the statistical values to characterize the increasing degree of microhardness (figure 16). When given the scanning speed 10 mm/s, the beam diameter 3 mm and the laser power 1200W (S1, figure 14), 1300W (S2, figure 14), 1400W (S3, figure 14) and 1500W (S4, figure 14), the cladding layer has significantly larger microhardness than the substrate, exhibiting the increasing rates of 211.0, 166.4, 164.6 and 155.3%, respectively. This indicates that the cladding layer (Ni60/WC) improves the microhardness of ductile iron considerably.

By analyzing the results from simulation and experiment, we determined the laser cladding parameters that causes the cladding layer to have a relatively better quality. The cladding parameters of scanning speed 10 mm/s, beam diameter 3 mm and laser power 1200W, 1300, 1400 and 1500W make the molten pool exhibit appropriate temperature, and cause the cladding layer to have relatively smaller residual stress, almost no powder inclusions and favourable dilution rate. The cladding layers fabricated under the laser power 1200 and 1300W, scanning speed 10 mm/s and beam diameter 3 mm exhibit obvious pores and powder inclusions (figure 14a,b), and the cladding layer fabricated under the laser power 1500W, scanning speed 10 mm/s and beam diameter 3 mm has obvious pores, powder inclusions and microcracks (figure 14d). Whereas the cladding layer fabricated under the laser power 1400W, scanning speed 10 mm/s and beam diameter 3 mm exhibits relatively better morphology, as without powder inclusions and with only a few tiny pores (figure 14 c). In terms of dilution rate, the four types of cladding parameters cause the cladding layers (S1~S4) to exhibit appropriate values (11.29~13.41%, table 1), which satisfy the requirements of high-quality cladding layer. In microhardness, the four types of cladding parameters make the cladding layers (S1~S4) exhibit much larger microhardness (increase rate 155.3~211.0%, figure 16). Therefore, the laser cladding parameters of laser power 1400W, scanning speed 10 mm/s and beam diameter 3 mm are the optimal combination for acquiring the cladding layer with high quality, satisfying the requirement for the laser cladding repair of uneven wear in brake discs from heavy-duty vehicles.

## 5. Conclusions

In summary, by the simulation and experiment, we investigated the influence of laser cladding parameters on the quality of cladding layer for repairing brake discs in heavy-duty vehicles. Laser cladding parameters have a significant effect on the thermal properties of the molten pool. Appropriate parameters cause the molten pool to exhibit favourable temperature, and the cladding layer to have relatively small residual stress, almost no powder inclusions and beneficial dilution rate. The obtained cladding layers (Ni60/WC) exhibit significantly larger microhardness than the substrate (ductile iron), as the increase rate ranges from 155.3 to 211.0%. The cladding parameters of laser power 1400W, scanning speed 10 mm/s and beam diameter 3 mm are the optimal combination for acquiring the cladding layer with a relatively better quality. An available reference has been offered for the laser cladding repair of uneven wear in heavy-duty vehicles’ brake discs.

## Data Availability

All original data have been submitted to the Dryad [34].
